# Long-Term Quality of Life (BREAST-Q) in Patients with Mastectomy and Breast Reconstruction

**DOI:** 10.3390/ijerph18189707

**Published:** 2021-09-15

**Authors:** Silvia García-Solbas, Miguel Ángel Lorenzo-Liñán, Gracia Castro-Luna

**Affiliations:** 1 Department of Obstetrics and Gynaecology, Hospital Vithas Virgen del Mar, 04120 Almería, Spain; 2 Department of General and Digestive Surgery, University Hospital Torrecardenas, 04009 Almería, Spain; malorenzol@hotmail.com; 3 Department of Nursing, Physiotherapy and Medicine, University of Almería, 04120 Almería, Spain; graciacl@ual.es

**Keywords:** breast cancer, breast cancer risk-reducing surgery, mastectomy, breast reconstruction, quality of life, BREAST-Q

## Abstract

(1) Background: Mastectomy is the surgical treatment of choice in 20–30% of women with breast cancer. In addition, more women are undergoing risk-reducing mastectomies. It is necessary to study these women’s quality of life and satisfaction after surgery, as studies report high percentages of dissatisfaction with the results. The publication of the BREAST-Q© questionnaire in 2009 provided a valuable tool to measure these results. (2) Methods: Descriptive, cross-sectional study of 70 patients who underwent mastectomy and breast reconstruction, both therapeutic and prophylactic, in the last 10 years to whom the BREAST-Q© 2.0-Reconstruction Module questionnaire was provided for completion. (3) Results: The sexual satisfaction scale was the lowest score of the entire questionnaire (51.84 ± 21.13), while the highest score was obtained on the satisfaction with the surgeon scale (91.86 ± 18.11). The satisfaction with care scales showed the importance of the evaluation of these items for future studies. More than half of the patients of the study (51.5%) underwent at least one reoperation after the first surgery, with an average of one (1.15) intervention per patient and a maximum of five. (4) Conclusions: Mastectomy and breast reconstruction have a high negative impact on the sexual well-being of patients. The high percentage of reoperations is a factor to consider because of its possible influence on these patients’ quality of life and satisfaction.

## 1. Introduction

Breast cancer is the most frequent malignant neoplasm in women. In 2020, there were 2.3 million women diagnosed with breast cancer and 685,000 deaths globally. As of the end of 2020, there were 7.8 million women alive who were diagnosed with breast cancer in the past 5 years, making it the world’s most prevalent cancer. There are more lost disability-adjusted life years (DALYs) by women to breast cancer globally than any other type of cancer [1]. In the U.S., in 2021, an estimated 281,550 new cases of invasive breast cancer are expected to be diagnosed in women in the U.S., along with 49,290 new cases of non-invasive (in situ) breast cancer.

About 43,600 women in the U.S. are expected to die in 2021 from breast cancer. Death rates have been steady in women under 50 since 2007, but have continued to drop in women over 50. The overall death rate from breast cancer decreased by 1% per year from 2013 to 2018. These decreases are thought to be the result of treatment advances and earlier detection through screening [2].

Recently Miller et al. analyzed population-based cancer incidence and mortality for AYAs in the U.S. by age group (ages 15–19, 20–29, and 30–39 years), sex, and race/ethnicity. In 2020, it was estimated approximately 89,500 new cancer cases and 9270 cancer deaths in AYAs [3].

In Spain it represents 36% of tumors in women with almost 33,000 new cases per year. It is the leading cause of death from cancer in Spanish women, although, since the 1980s, there has been a downward trend in mortality. This is due to the combined effect of screening programs, improved treatments, and multimodal therapy [4].

Despite the significant advances in tumor biology in recent years, surgery remains an essential part of the primary treatment of breast cancer, although this has also evolved over the years. Nowadays, it is possible to diagnose the tumor in its early stages, which allows conservative surgery to be performed, thus minimizing the esthetic impact, as long as the oncologic conditions allow it. However, conservative techniques are not always possible, so mastectomy remains a frequent procedure, and approximately 20–30% of women with breast cancer undergo it [5].

On the other hand, recent discoveries in the genetic basis of breast cancer have led to increased interest in risk-reducing mastectomy as a method of breast cancer prevention [6,7].

Over the years, surgical strategies have changed. Although the primary objective of treatment continues to be disease control, improved cosmetic results have become more critical to reduce patients’ physical and psychological trauma. Thus, according to the Halsted technique, mastectomy was replaced by modified radical mastectomy; the objective has been to produce the aggression as atraumatic as possible. To this end, skin-sparing mastectomy, where most of the skin envelope is preserved, and skin- and nipple-sparing mastectomy, where, in addition, the nipple-areola complex can be preserved, have emerged allowing better esthetic results after post-mastectomy reconstruction [8].

In addition, the increased survival of breast cancer patients has led to greater attention to their quality of life. It is why breast reconstruction is now a necessary part of the overall treatment. It has been shown not to influence patient survival [9] and helps restore body image and relieve the stress associated with mastectomy [10].

Post-mastectomy reconstruction can be performed immediately or deferred. There are various procedures for performing it (tissue expansion-prosthesis; autologous tissue), the choice of which will depend on the patient’s characteristics, thus allowing the most personalized treatment possible to be obtained [11].

Nevertheless, choosing the type of breast reconstruction is a difficult decision and optimizing the results of breast cancer surgery remains a challenge. Approximately 40% of women who undergo surgery reported dissatisfaction with their surgery’s decision and esthetic results [12,13]. Some authors such as Shechter et al. reported that patients in the breast reconstruction group had higher scores for esthetic outcome and a higher degree of patient satisfaction from the surgical outcome compared to the patients in the breast conservating treatment-alone group [14].

Lei et al. in a multi-center study identified factors of higher patient satisfaction, such as NAC preservation and absence of radiotherapy, in order to help breast surgeons make better decisions about individualized reconstruction plan [15].

Schmidt et al. reported that patients who found their breast to be highly important were more likely to decide for a reconstruction. For these authors patients reporting a high significance of their breast showed the greatest decrease in satisfaction with their breast after reconstruction [16].

Therefore, the objective of this surgery, beyond the final esthetic result, must consider how it will affect the patient’s quality of life and satisfaction. Thus, these variables have become a fundamental part of evaluating the success of breast surgery [17].

In order to measure these variables of effect, we have questionnaires based on the results reported by the patients themselves (“Patient-reported outcomes-PROs”). These instruments quantify aspects related to health-related quality of life (HRQOL), such as, for example, satisfaction; obtaining the information directly from the patient themself without any interpretation by third parties; and, in addition, they include broader concepts such as satisfaction with the care received [18].

Multiple instruments have been used to measure PROs in breast surgery patients. However, until just over a decade ago, few of these questionnaires had sufficient evidence to be used specifically in these patients, as they had limitations in certain areas, such as those related to esthetics and body perception [19].

In 2009, the BREAST-Q questionnaire was developed to meet this need, a validated PRO instrument specific to breast surgery. Since its release, it has substantially improved our ability to study the results of this procedure from the patient’s point of view and patient satisfaction [20].

Drs. Pusic, Klassen, and Cano developed the BREAST-Q© questionnaire, owned by Memorial Sloan-Kettering Center and Columbia University (U.S.). It has been available for use in Spain since 2016 after linguistic validation, and in 2017 they launched version 2.0, which was tested on a much larger group of patients. It consists of six modules; two modules related to esthetic purposes (breast augmentation and reduction) and the breast cancer module, which encompasses the mastectomy, breast reconstruction, reconstruction expectations, and breast-conserving surgery modules. All modules are based on a conceptual structure with six domains that are grouped into two: the first domain related to the quality of life that included: physical well-being, psychological well-being, and sexual well-being. Moreover, the second domain is related to patient satisfaction (the breasts, the results, and the medical care received). Each module contains preoperative and postoperative scales, which can be used independently or together to measure changes [21].

Since the appearance of the BREAST-Q© questionnaire, many studies have used it to evaluate breast surgery patients. However, these studies have identified limitations, such as short follow-up periods or small sample sizes [13,22]. In contrast, other studies, although extensive, are only limited to therapeutic reconstruction techniques, excluding other techniques from the study [14,23,24]; or they are limited to patients who underwent breast cancer surgery, excluding risk reduction surgeries [25].

The objective has been to evaluate the satisfaction and quality of life in women who have undergone mastectomy with breast reconstruction, compare the esthetic results, and identify risk factors that may influence these results.

## 2. Materials and Methods

### 2.1. Study Design

This study is a descriptive cross-sectional study of women who underwent mastectomy surgery with reconstruction and were submitted to a questionnaire of satisfaction and quality of life specific to breast reconstructive surgery (BREAST-Q Version 2.0—Reconstruction module, validated in Spanish).

### 2.2. Study Population

Patients undergoing mastectomy with breast reconstruction from 2010 onwards, at the Hospital Universitario Torrecárdenas and in private clinics in Almería, operated on by the same surgical team, met the following selection criteria:

- Women with breast cancer with an indication for a mastectomy followed by reconstruction.
- Women with breast cancer who refuse conservative treatment.
- Women undergoing uni- or bilateral risk reduction surgery followed by reconstruction.
- Who has undergone surgery at least 12 months before the time of inclusion in the study.
- Who sign informed consent for inclusion in the study.

Exclusion criteria were:

- Women with early metastatic disease (stage IV).
- Women with inflammatory carcinoma.

### 2.3. Sample Size

Patients who had undergone mastectomy with breast reconstruction since 2010 operated on at the Hospital Universitario Torrecárdenas and in private clinics in the province of Almería by the same surgical team and met the criteria for inclusion in the study were selected. The type of sampling was by convenience through access to the patients’ medical records.

The sample size was calculated based on a series of estimates considering the latest data recorded on the number of patients diagnosed with breast cancer and undergoing mastectomy in the province of Almeria during the year 2020, whose figure was 111 patients.

For a finite population, the Ene 3.0 statistical program (UAB, Barcelona, Spain) was used to obtain the sample size, assuming an alpha error of 0.05, an expected proportion of 50%, and a precision of 5%, obtaining an estimated sample of 70 patients.

### 2.4. Data Collection and Sources of Information

After signing the corresponding informed consent, the patients selected during the period included in the study were invited to participate in the study by filling in the questionnaires referred to above, with at least one year having elapsed since the surgical intervention. A series of pre-and post-surgical variables of a demographic nature and relating to the characteristics of the tumor, surgical intervention, complications and adjuvant treatments, obtained from the patient’s clinical history, were also collected.

### 2.5. BREAST-Q© Questionnaire

The questionnaire is a PRO tool designed to evaluate the results of women undergoing different types of breast surgery. Drs. Pusic, Klassen, and Cano developed it and it is owned by Memorial Sloan-Kettering Cancer Center and Columbia University (U.S.). Version 1.0 of the questionnaire was published in 2009 and has been available in Spain since 2016 after linguistic validation. In 2017, version 2.0 of the questionnaire was published, which is the one currently used.

The questionnaire contains six modules, of which the first two are related to cosmetic surgery and the rest to breast cancer surgery:

1. Breast augmentation;
2. Reduction/mastopexy;
3. Breast cancer;
  - Mastectomy;
  - Reconstruction;
  - Breast reconstruction expectations;
  - Breast-conserving surgery.

Each module, in turn, is divided into multiple scales that can be used independently, allowing each researcher to generate a questionnaire tailored to his or her research. In addition, each module has preoperative and postoperative scales, which can be used individually or together, allowing changes over time to be assessed.

All modules are based on a conceptual structure comprising six domains, grouped into the two described below. In addition, due to the importance of body image for breast surgery patients, numerous questionnaire scales allow the measurement of this concept.

#### 2.5.1. Health-Related Quality of Life

- Psycho-social well-being: this scale contains items that inquire into the patient’s body image (e.g., body acceptance, feeling of being attractive) and her confidence in certain social situations;
- Sexual well-being: Its items are intended to measure the patient’s sexual well-being through questions about sensations when she is dressed or naked, her sexual confidence concerning the appearance of her breasts and her sensations during sexual activity;
- Physical well-being: This scale differs between the different modules. Depending on which one we are in, we will see questions relating to physical problems in the chest, back, abdomen or skin due to the adverse effects of radiotherapy.

#### 2.5.2. Patient Satisfaction

- Breast Satisfaction. This scale measures body image through patients’ satisfaction with their breasts, based on questions about the comfort of the operated or reconstructed breasts, both clothed and unclothed. It also evaluates symmetry, breast smoothness, and some items related to implants in the reconstruction module;
- Satisfaction with the results. It includes items related to the patient’s satisfaction with the appearance after surgery of the nipple-areola complex, especially regarding the scars;
- Satisfaction with the care received. It is a scale that only this questionnaire has and which makes it possible to evaluate the patients’ satisfaction with several points of their surgical process: the information received from their surgeon on the procedures and recovery times, satisfaction with the surgeon (professionalism, sensitivity, communication skills, accessibility), satisfaction with the care received by the rest of the medical team and satisfaction with other non-medical personnel involved in the process (for example, administrative personnel).

As mentioned above, it is not necessary to complete all the modules, but they are completed independently according to the purpose of each study. Brief instructions are given at the beginning of each scale to allow the patient to understand the items better. In addition, the questionnaire user’s guide expressly states that its use is permitted both in printed format and by creating its online version to facilitate its administration to the study subjects 88.

An overall score is not obtained for the questionnaire; each scale is scored independently. All scores fluctuate between 0 and 100, so that a higher score implies greater satisfaction or better quality of life.

Finally, since the questionnaire is the property of Memorial Sloan-Kettering Cancer Center and Columbia University, in order to use it for research purposes, a license for its use must be obtained, free of charge, through their official website (http://qportfolio.org/breast-q/(Accessed on 4 January 2021)).

### 2.6. Study Variables

#### 2.6.1. Independent Variables

- Age (at the time of surgery): quantitative variable expressed in years;
- Body mass index: quantitative;
- Tobacco use YES/NO: dichotomous qualitative variable;
- Educational level (Low/Medium/High): ordinal qualitative variable;
- Previous surgery (No/Benign surgery/Conservative surgery for cancer + Radiotherapy/Augmentation surgery/Reduction-pexy): qualitative variable;
- Reason for the intervention (Cancer/High risk/BRCA/BRCA + cancer);
- Tumor location within the breast: qualitative variable;
- Tumor size (mm): quantitative variable, expressed in millimetres;
- Tumor extension (Single/Multifocal/Multicentric/Extensive): qualitative variable;
- Molecular subtype (Luminal A/Luminal B/Triple positive/Her two neu overexpressed/Basal-like): qualitative variable;
- Tumor pathologic TNM (Tis/T1/T2/T3): ordinal qualitative variable;
- Nodal pathologic NCT (N0/N1/N2): ordinal qualitative variable;
- Tumor stage (0/I/IIA-B/IIIA-B): ordinal qualitative variable;
- Date of surgery;
- Type of surgery: classic mastectomy/skin-sparing mastectomy type I-II/skin-sparing mastectomy type IV/skin- and nipple-sparing mastectomy/partial + dorsal mastectomy): qualitative variable;
- Timing of breast reconstruction (Immediate/Different): dichotomous qualitative variable;
- Type of reconstruction (Expansion-prosthesis/Becker-type expander/direct implant/dorsal-prosthesis/biological mesh): qualitative variable;
- Treatment of the axilla (No/BSGC/axillary lymphadenectomy): qualitative variable.
- Implant size: quantitative variable expressed in grams;
- Chemotherapy (No/Neoadjuvant/Adjuvant): qualitative variable;
- Radiotherapy (No/Over expander/Over prosthesis/Over Becker/Over flap): qualitative variable;
- Contralateral breast symmetrization (NO/Augmentation/Reduction/Pexia + Implant/Preventive mastectomy): qualitative variable.

#### 2.6.2. Dependent Variables

- Immediate complications (No/Seroma/Wound dehiscence/Skin or flap necrosis/CAP necrosis/Implant extrusion): qualitative variable;
- General evolution after the first intervention (Favorable/Reoperation): qualitative dichotomous variable;
- Late complications of breast reconstruction (Satisfactory/Discomfort/Asymmetry/Contracture III-IV/Discomfort + Asymmetry/Implant rupture/Delayed seroma/Others): qualitative variable;
- Post-surgical complications of the symmetrized breast (NO/YES): dichotomous qualitative variable;
- Number of reoperations: discrete quantitative variable;
- Oncologic outcome (Disease-free/Systemic recurrence/Local recurrence/Exitus): qualitative variable;
- Date of completion of the questionnaire;
- Time elapsed between breast reconstruction and completion of the questionnaire in years: quantitative variable;
- Time elapsed between breast reconstruction and completion of the grouped questionnaire (1–3 years/4–6 years/7–9 years/10 years): qualitative variable;
- Results of the BREAST-Q questionnaire version 2.0 Post-surgical reconstruction module.

### 2.7. Ethical Implications

The project complies with the ethical principles of human research in the Declaration of Helsinki and the Biomedical Research Act. Likewise, it complies with the stipulations of the Organic Law 3/2018, of 5 December, on Personal Data Protection and guarantee of digital rights, guarding its strict confidentiality and its non-access to unauthorized third parties, as well as with the European Union Regulation 2016/679. Each patient who wished to participate was provided with a copy of the patient information sheet and informed consent. The Hospital Torrecárdenas Ethical Committee approved the study Code number: 47/2021.

### 2.8. Statistical Analysis

The study variables and questionnaire scores will be collected in a database and subsequently analyzed with IBM SPSS 26 (IBM Corp. Released 2019.Version 26.0. IBM Corp, Armonk, NY, USA).

A descriptive analysis of the main variables will be performed. For qualitative variables, their frequency distribution (number of cases and percentage) will be obtained. Quantitative variables will be expressed as a minimum, maximum, mean and standard deviation. Before performing the bivariate analysis, normality tests will be performed for quantitative variables using the Kolmogorov-Smirnov test. Parametric tests (Pearson’s correlation, t-Student, and ANOVA) will be used for those following a normal distribution and non-parametric tests (Spearman’s correlation, Mann Whitney U, and Kruskal-Wallis) for those not following a normal distribution.

## 3. Results

### 3.1. Descriptive Statistics

Seventy participants were included in the study, of which 50 returned the questionnaire correctly completed (71.42%). The most relevant demographic and preoperative characteristics of the study sample are described in Table 1 and Table 2. The mean age of the study population was 45.94 (7.85) years, and the mean time elapsed between breast reconstruction and completion of the questionnaire was 4.52 (3.22) years

Concerning the qualitative variables studied, we found that 24.2% of the patients studied were smokers; 43.6% had a high level of education, and 25% of them had previously undergone surgery on the affected breast, either for esthetic reasons or for benign pathology.

In terms of tumor characteristics, the most frequent tumor location was the upper outer quadrant in 56.1% of cases, and 62.1% of patients were stage II A/B at the time of surgery.

The most important characteristics of surgery, adjuvant therapy and evolution of patients of study are described in Table 3. The most frequently performed intervention was skin-sparing mastectomy type I/II in 44.1% of the patients. Immediate post-mastectomy reconstruction was carried out in 94.1% of the patients in the same surgical act, the most frequently used technique being reconstruction with alloplastic tissue, with a two-stage expander-prosthesis in 30.9% of cases, followed by reconstruction with a Becker-type expander (25%) and reconstruction with a latissimus dorsi muscle flap (25%). A total of 57.4% of the patients underwent surgery to symmetrize the contralateral breast, either in the same surgical procedure or at a second stage. This surgery was a preventive mastectomy in 16.2% of them.

In terms of adjuvant treatment, 39.7% of the patients studied received radiotherapy on the reconstructed breast, while 64.7% of them received chemotherapy (26.5% neo-adjuvant chemotherapy and 38.2% adjuvant chemotherapy).

We also analyzed the evolution of the patients after the first surgery. A total of 51.5% of them had to undergo at least one second operation, with the maximum number of reoperations found being five. A total of 19.1% suffered some type of immediate complication after surgery, the most frequent being skin necrosis in 5.9% of cases. A total of 56.7% of the patients suffered a late complication that forced them to undergo reoperation, the most frequent complication being Baker’s grade III–IV capsular contracture in 44.8% of cases.

The mean scores obtained in the scales studied in the BREAST-Q questionnaire are described in Table 4. The scale with the lowest score is sexual well-being, with a mean of 51.84 (21.13) points; the scales with the highest scores are those evaluating satisfaction with the care received: satisfaction with the surgeon with a score of 91.86 (18.11), satisfaction with the surgical team with a mean of 88.58 (20.06), and satisfaction with the hospital with 81.8 (23.63) points.

### 3.2. Differential and Bivariate Analyzes

#### 3.2.1. Health-Related Quality of Life Scales

Table 5 shows the differences obtained between the main scores obtained in the health-related quality of life scales and the main variables studied.

When comparing the psycho-social well-being scale score according to other factors related to the patients and their intervention, we found a statistically significant association (*p* = 0.01) between the score on this scale and the history of previous surgery on the affected breast. The mean score among patients with some previous surgery was 52.54 (19.16) points, compared to 69.16 (20.59) points in those with no previous history. This association was also found when comparing the mean score of the sexual well-being scale with this personal history of the patient, finding a mean score of 56.03 (19.50) points among patients with no history of surgery, compared to 39.92 (21.75) points among those with previous surgery (*p* = 0.01).

When comparing the mean scores of physical well-being according to the location of the primary tumor, we found a mean score of 70.78 (19.40) when the tumor was located in the superior external quadrant of the breast, compared to 39.60 (14.58) when it was located in the inner-internal quadrant, this difference being statistically significant (*p* = 0.01).

We found no statistically significant association between the mean score on the three quality of life scales and the timing or type of breast reconstruction performed.

Table 6 describes the results obtained by correlating the score on the health-related quality of life scales with the different quantitative variables studied. We found no statistically significant association between the mean score in psycho-social well-being with any of them.

When comparing the mean score of the sexual well-being scale with the quantitative variables, we found an inverse correlation between the score on this scale and the patient’s age (r = −0.35; *p* = 0.01) without finding an association with the rest of the variables studied.

Finally, we found no statistically significant relationship between the mean score of physical well-being with the quantitative variables studied (Table 5).

#### 3.2.2. Patient Satisfaction

Table 7 shows the differences between the mean scores in patient satisfaction scales and the qualitative variables studied, and Table 8 shows the results after correlating the mean score on both scales with the quantitative variables of the study.

No statistically significant association was found between mean score in satisfaction with breast scale with any of the variables studied, both quantitative and the rest of qualitative variables.

NAC reconstruction was performed in 33 of the patients surveyed. Of these, 45.5% reported being dissatisfied with the results. We found a statistically significant inverse relationship between the mean score on satisfaction with NAC reconstruction scale and the years since surgery, with a correlation coefficient of −0.40 (*p* = 0.02).

Evaluating the rest of the qualitative variables, we found no statistically significant differences between the mean scores on the scale. Although there was a difference in the mean score according to the type of mastectomy: skin-sparing mastectomy type I–II had a mean of 1.91 (1.13), compared to the skin-nipple-sparing mastectomy with a mean of 3.22 (0.97) and skin-sparing mastectomy type IV whose mean was 2.80 (0.83). However, these differences did not reach statistical significance (*p* = 0.07).

The rest of the scales studied (satisfaction with the information received, satisfaction with the surgeon, satisfaction with the medical team and satisfaction with the hospital) are included in the care received. Table 9 shows the differences obtained between the mean scores in those scales and most important variables studied.

Concerning the satisfaction scale with the information received, we found differences in the mean score depending on when the breast reconstruction was performed. In those cases where immediate reconstruction was performed, the mean score was higher with a mean of 68.80 (18.69), compared to those with delayed reconstruction, whose mean was 52.50 (8.02), this difference being statistically significant (*p* = 0.04). No relationship was found between the mean score of this scale and the rest of the variables studied.

The mean score on the surgeon satisfaction scale was associated with the patient’s tumor stage at diagnosis. Women with stage II A-B had a mean score of 95.07 (9.98) points compared to those with stage 0, whose mean score was 43 (60.81) points; this difference was statistically significant (*p* = 0.04). The score on this scale was also associated with the type of breast reconstruction performed. Patients who underwent direct implant reconstruction had a mean score on this scale of 100 points compared to 85.90 (17.34) points in those who underwent latissimus dorsi flap reconstruction. This difference is statistically significant (*p* = 0.04).

Finally, the mean scores on the scales of satisfaction with the surgical team and satisfaction with the hospital were associated with the patient’s level of education. Patients with a high level of education scored higher on both scales than those with a low level of education. In the case of the satisfaction with the surgical team scale, the mean score in the highly educated patients was 96.44 (9.93) points compared to 72.33 (32.91) points in the low educated group (*p* = 0.02). On the hospital satisfaction scale, the mean score was 91.75 (14.16) points in the high-level group versus 60.89 (27.32) points in the low-level group (*p* = 0.003).

## 4. Discussion

The development of the BREAST-Q questionnaire in 2009 laid the groundwork for assessing the satisfaction and quality of life of women undergoing breast surgery with a validated tool that allows the measurement of patients’ perceptions. Since its publication, it has become the gold-standard PRO instrument used in breast surgery.

In addition to its excellent metric properties, since it is a self-administered questionnaire, which can even be filled out online, it facilitates its completion by the patient independently, which verifies her answers. In our study, of the 70 participants included, 71.42% returned the questionnaire correctly completed, this response rate being in line with that found in other studies [26].

### 4.1. Health-Related Quality of Life

In the three scales related to health-related quality of life (HRQOL): psycho-social well-being, sexual well-being and physical well-being, we found no association with the patient’s body mass index, while other similar studies have found that. These results are surprising since the relationship between BMI and HRQOL has already been extensively studied, finding that the effect of BMI on HRQOL is independent of disease severity [27]. In contrast, other studies have shown that obesity is associated with worse outcomes and poorer survival in women with breast cancer [28].

Regarding sexual well-being, it is the scale with the worst mean score of the entire questionnaire, which corresponds to that found in the literature when compared with other similar studies [23,29,30]. The negative relationship between age and sexual well-being is widely described in other studies, so it does not seem to be an association specific to mastectomy patients [31]. Multiple studies have evaluated sexual well-being in patients after breast cancer, some of which report that sexual dysfunction can affect up to 90% of women treated for breast cancer [32]. These problems are generally associated with the disturbance suffered in the patient’s body image after surgery [33], constituting an essential long-term side effect of the treatments used [34]. In particular, sexual dysfunction in body image worsens in women treated with mastectomy compared to conservative treatment [35]. Furthermore, an impact on sexuality has also been demonstrated in women who undergo prophylactic mastectomy, so this should be a side effect to consider and inform patients who request this type of risk-reducing surgery [36].

Our study found no statistically significant differences between the patients’ quality of life in the three domains studied (psycho-social, sexual, and physical well-being) and the time and type of breast reconstruction performed. These results differ from what has been found so far in the literature. A systematic review published in 2018 by Liu et al. included 54 studies evaluating the quality of life and satisfaction of patients who underwent breast oncoplastic surgery using the BREAST-Q questionnaire. The authors found higher scores in the three HRQOL domains, comparing immediate reconstruction with delayed reconstruction and autologous tissue versus reconstruction with implants [37]. In our study, the lack of significance is most likely due to the small sample size, in which 92.2% of the patients underwent immediate reconstruction.

### 4.2. Satisfaction with the Results

This domain of the BREAST-Q questionnaire includes the scales of satisfaction with the breasts, the implants, the reconstruction of the nipple-areola complex (NAC), and the care received.

The study did not find a statistically significant relationship with the factors studied regarding satisfaction with the breasts. Differences were observed between the mean scores on the scale according to the type of mastectomy. However, they almost reached significance (*p* = 0.07), finding that skin-sparing mastectomy type IV and skin- and nipple-sparing mastectomy obtained slightly higher scores than classic mastectomy and skin-sparing mastectomy type I-II. Compared with the existing literature in this regard, Nelson et al. in a study published in 2019 reported lower breast satisfaction in classic mastectomy compared to the rest of the techniques. In addition, in that study, lower breast satisfaction was associated with other factors such as having received postoperative chemotherapy or radiotherapy and non-symmetrization of the healthy breast [24]. By increasing the sample size of our study, we expect to find more relevant results related to this scale.

This study did not find a relationship between satisfaction with the results studied (breast, implants, and NAC reconstruction) and postoperative radiotherapy. Studies on this subject show an increase in morbidity in all forms of breast reconstruction in the context of post-surgical radiotherapy [38]. The meta-analysis reported a complication rate of four times higher than breast reconstructions that had not undergone radiotherapy afterwards. The most frequent complications were capsular contracture, pain, and distortion of the implant [38,39].

Nipple-areola complex (NAC) reconstruction was performed in 33 of the patients surveyed. Of these, 45.5% reported being dissatisfied with the results, which shows that, despite the potential physical and psychological impact on the patient performing this last step of breast reconstruction, the results obtained reduce patient satisfaction. We found a statistically significant inverse relationship between the mean score on this scale and the years since surgery, with a correlation coefficient of −0.40 (*p* = 0.02). On the other hand, we did not find a statistically significant association between the type of surgical technique used to perform the mastectomy and satisfaction with the reconstruction of the NAC. This result differed from the findings of other studies showing that nipple-skin-sparing mastectomy is a more accepted technique because it preserves body integrity and reduces the feeling of mutilation. In addition, preserving the NAC from the beginning does not require specific reconstruction of the NAC, so the esthetic results are better [40].

### 4.3. Satisfaction with the Results

The subscales included in the satisfaction with the care received are the most neglected of the BREAST-Q questionnaire. At the same time, they are a strong point of this questionnaire, as no others take these aspects into account [41]. The studies have shown that the patient’s subjective evaluation of the results after breast reconstruction considers both the cosmetic results and the trust placed in the medical team and the feeling of being cared for by them [42]. The surgeon–patient relationship has a significant influence on the results perceived by the patients [43].

High scores were obtained on the scales relating to satisfaction with the surgeon, the surgical team, and the hospital. Although in the scale related to satisfaction with the information received, a lower mean score of 67.47 (18.56) points was obtained. This distribution of scores on these scales agrees with what has been found in the literature to date [13,30].

As for the factors that could influence satisfaction with the care received, the most striking association was found between the patient’s level of education and satisfaction with the surgical team and the hospital; the lower the level of education, the lower the satisfaction with the two scales.

Finally, an essential point of our study was to analyze the evolution of the patients after surgery, finding that 51.5% of them had to undergo at least one-second operation. A Canadian study published by Roberts et al. reported a mean reoperation rate after the first reconstruction surgery of two operations over a 5-year follow-up period [44]. In our study, the mean number of reoperations per patient was 1.00 (1.15) with a maximum of up to 5 operations. Another study published by Boughey et al. showed a strong association between the rate of reoperations and lower overall patient satisfaction [45]. In our study, we found no association between the number of reoperations and other satisfaction scales of the questionnaire. However, this may be due to the small sample size, so we will continue this line of research in future studies.

## 5. Strengths and Weakness of the Study

The main limitation of the study is the reduced sample size of 70 patients, although the calculated sample size was met, as reflected in the methodology, for the population studied and other similar studies have even smaller sample sizes.

The type of sampling by which the study sample was chosen is a convenience sample, a non-probabilistic and non-random sample, as the patients were chosen because of the ease of access to their medical records, which means that it may not be a representative sample of the population. In addition, by selecting patients operated on by the same surgical team there may be a selection bias, although this is also a strength of the study, as having been operated by the same team the surgical techniques are more comparable, and so are the results.

Patients were followed up for up to 10 years, which is one of the strengths of the study. However, with such a long study period, it is possible that some patients may not be contacted, or that some may have died, so that data will be lost.

Finally, another potential limitation of our study is that we do not know the baseline status of the patients in terms of their well-being and satisfaction. Since the BREAST-Q© questionnaire also has preoperative scales, it would be interesting for future studies to analyze the state of the patients before surgery in order to better assess their changes.

## 6. Conclusions

The BREAST-Q© questionnaire was confirmed as a suitable tool that is easy to administer and complete to assess the quality of life and satisfaction of patients who underwent mastectomy and breast reconstruction.

The sexual well-being scale is the worst scored of all the questionnaires, which confirms the negative impact that mastectomies have on body image and sexuality, even those performed prophylactically. Reconstruction of the nipple-areola complex often leads to decreased patient satisfaction with the esthetic results, contrary to the initial objective of reconstructive surgery, which is to improve the sensation of mutilation and its impact on body image. More than half of the patients in the study had to undergo at least a second surgery after reconstruction, so it is essential to consider this rate because of its possible influence on the quality of life and patient satisfaction.

Finally, evaluating satisfaction with medical care is as critical as evaluating cosmetic results since the surgeon-patient relationship can affect overall satisfaction with the process.

## Figures and Tables

**Table 1:** Demographic characteristics and preoperative conditions (I).

	Minimum	Maximum	Mean	Std. Deviation
Age at diagnosis	26	64	45.94	7.85
BMI *	17.72	35.16	24.85	3.58
Tumor Size (mm)	5	75	31.44	17.06
Implant Size (cc or grs)	200	560	370.9	80.71
Number of reoperations	0	5	0.99	1.15
Years from mastectomy to questionnaire completion	1	10	4.52	3.22

* BMI = Body Mass Index.

**Table 2:** Demographic characteristics and preoperative conditions (II).

Variable	*n*	%
Smoking		
Yes	15	24.2%
No	30	75.8%
Education level		
Primary education	9	23.1%
Secondary education	13	33.3%
University degree	17	43.6%
Previous breast surgery		
Yes	17	25%
No	51	75%
Localization of primary tumor		
Upper-outer quadrant	37	56.1%
Upper-Inner quadrant	9	13.6%
Lower-outer quadrant	4	6.1%
Lower-Inner quadrant	7	10.6%
Central	9	13.6%
Stage		
0	2	3%
I	11	16.7%
IIA-IIB	41	62.1%
IIIA-IIIB	12	18.2%

**Table 3:** Characteristics of surgery, adjuvant therapy and evolution of patients.

Variable	*n*	%
Type of mastectomy		
Skin-sparing type I–II	31	45.6%
Skin-sparing type IV	8	11.7%
Nipple skin-sparing	10	14.7%
Radical mastectomy	19	28.0%
Breast reconstruction timing		
Immediate	64	94.1%
Delayed	4	5.9%
Type of reconstruction		
Expander-prosthesis	21	30.9%
Becker expander	17	25%
Latissimus dorsi muscle flap	17	25%
Matrix acellular	6	8.8%
Direct prosthesis	7	10.3%
Lymph node management		
None	2	2.9%
Sentinel lymph node biopsy	39	57.4%
Axillary lymph node dissection	27	39.7%
Adjuvant Radiotherapy		
Yes	27	39.7%
No	41	60.3%
Chemotherapy		
No	24	35.5%
Neo-adjuvant	18	26.5%
Adjuvant	26	38.2%
Breast symmetrisation		
Yes	39	57.4%
No	29	42.6%
Evolution after first surgery		
Successful	33	48.5%
Reoperation	35	51.5%

**Table 4:** Results of the BREAST -Q questionnaire.

Breast-Q Scales	Minimum	Maximum	Mean	Std. Deviation
Psychosocial well-being	18	100	64.84	21.35
Sexual well-being	14	100	51.84	21.13
Physical well-being	14	100	62.83	22.11
Satisfaction with surgical results	0	100	55.86	16.84
Satisfaction with information	30	100	67.47	18.56
Satisfaction with the surgeon	0	100	91.86	18.11
Satisfaction with the surgical team	0	100	88.58	20.06
Satisfaction with the hospital	0	100	81.8	23.63

**Table 5:** Differences between health-related quality of life scales mean scores and qualitative variables studied.

Variables		Psycho-Social Well Being	*p*	Sexual Well-Being	*p*	Physical Well-Being	*p*
Smoking	Yes	57.07 (21.96)	0.16 *	46.67 (21.90)	0.33 *	58.14 (26.15)	0.49 *
	No	66.53 (20.86)		53.43 (22.05)		63.23 (21.11)	
Education level	Primary education	56.89 (20.11)	0.10 **	49.11 (16.78)	0.33 **	59.13 (29.55)	0.91 **
	Secondary education	55.83 (22.30)		45.75 (24.96)		58.67 (24.42)	
	University degree	62.97 (22.06)		58.62 (25.21)		62.25 (19.38)	
Previous breast surgery	Yes	52.54 (19.16)	0.01 *	39.92 (21.75)	0.01 *	53.62 (21.90)	0.07 *
	No	69.16 (20.59)		56.03 (19.50)		66.26 (21.48)	
Localization of primary tumor	Upper-outer	66.76 (20.87)	0.80 **	56.17 (19.97)	0.18 **	70.78 (19.40)	0.01 **
	Upper-inner	65.17 (31.08)		50.17 (27.48)		55 (28.69)	
	Lower-outer	57.67 (5.68)		49.33 (5.77)		48.33 (10.40)	
	Lower-inner	54.60 (20.18)		37.20 (17.62)		39.60 (14.58)	
	Central	62.20(24.37)		37.40 (18.54)		56 (22.27)	
Stage	0	67.50 (45.96)	0.98**	53.50 (7.78)	0.86 **	88.50 (4.95)	0.39 **
	I	63.00 (27.88)		50.78 (31.21)		59.78 (20.89)	
	IIA-B	64.86 (17.99)		49.32 (17.12)		60.63 (22.53)	
	IIIA-B	62.89 (24.45)		56.22 (22.90)		62.26 (22.40)	
Type of mastectomy	Skin-sparing type I–II	61.74 (22.24)	0.41 **	48.42 (22.10)	0.80 **	56.44 (20.50)	0.40 **
	Skin-sparing type IV	78.17 (13.36)		52.50 (10.34)		66.00 (14.47)	
	Nipple skin-sparing	66.00 (22.49)		56.67 (25.73)		71.22 (19.65)	
	Radical mastectomy	62.88 (21.85)		52.94 (21.27)		64.20 (27.02)	
Breast reconstruction timing	Immediate	65.41 (22.02)	0.52 *	52.15 (21.56)	0.72 *	63.55 (21.23)	0.46 *
	Delayed	58.25 (10.21)		48.25 (17.25)		55 (33.20)	
Type of reconstruction	Expander-prosthesis	61.87 (21.85)	0.18 **	43.73 (15.75)	0.21 **	60.20 (21.77)	0.57 **
	Becker expander	76.23 (15.81)		62.23 (18.59)		67.67 (19.11)	
	Latissimus dorsi muscle flap	57.60 (24.11)		48.70 (24.86)		67.67 (26.66)	
	Matrix acellular	55.40 (18.55)		50 (26.62)		65.60 (21.27)	
	Direct prosthesis	67.14 (23.12)		55.71 (23.21)		52 (23.66)	
Lymph node management	None	79.00 (2.82)	0.14 **	70.50 (28.99)	0.23 **	76.00 (5.65)	0.25 **
	Sentinel lymph node biopsy	59.38 (21.88)		47.85 (22.50)		58.00 (20.31)	
	Axillary lymph node dissection	70.00 (20.22)		54.86 (18.33)		67.33 (24.16)	
Adjuvant Radiotherapy	No	63.59 (19.89)	0.63 *	52.28 (21.48)	0.86 *	60.79 (21.80)	0.45 *
	Yes	66.57 (23.60)		51.24 (21.13)		65.70 (22.77)	
Chemotherapy	No	62.89 (23.17)	0.89 **	51.61 (24.18)	0.88 **	63.50 (19.35)	0.80 **
	Neo-adjuvant	65.69 (18.32)		54.23 (17.65)		59.17 (22.98)	
	Adjuvant	66.11 (22.44)		50.42 (21.17)		64.61 (24.93)º	
Breast symmetrisation	No	63.50 (21.82)	0.74 *	51.39 (25.37)	0.91 *	58.41 (24.57)	0.31 *
	Yes	65.59 (21.39)		52.09 (18.76)		65.26 (20.65)	
Evolution	Successful	67.00 (22.11)	0.53 *	53.64 (21.26)	0.59 *	63.33 (18.77)	0.89 *
	Reoperation	63.14 (20.97)		50.43 (21.29)		62.44 (24.73)	

* *p*-value obtained by t-Student; ** *p*-value obtained by ANOVA.

**Table 6:** Correlation between the score on the health-related quality of life scales and patient’s characteristics.

Variables		Correlation	*p*
Psycho-social well-being	Age	−0.15	0.27 *
	BMI	−0.26	0.06 *
	Tumor size	−0.22	0.13 **
	Implant size	−0.14	0.32 **
	Reoperations	0.08	0.55 **
	Years between surgery and the questionnaire	0.07	0.59 **
Sexual well-being	Age	−0.35	0.01 **
	BMI	−0.16	0.26 **
	Tumor size	−0.03	0.79 **
	Implant size	−0.20	0.15 **
	Reoperations	0.16	0.25 **
	Years between surgery and the questionnaire	0.05	0.70 **
Physical well-being	Age	−0.25	0.08 *
	BMI	−0.22	0.13 *
	Tumor size	−0.01	0.91 **
	Implant size	−0.01	0.90 **
	Reoperations	0.11	0.44 **
	Years between surgery and the questionnaire	0.25	0.07 **

* *p*-value obtained by Pearson’s correlation coefficient; ** *p*-value obtained by Spearman’s correlation coefficient.

**Table 7:** Differences between satisfaction with results scales mean scores and qualitative variables studied.

Variables		Satisfaction with Breasts	*p*	Satisfaction with the Reconstruction of the NAC	*p*
Smoking	No	54.37 (17.57)	0.83 *	2.64 (1.21)	0.10 ***
	Yes	55.47 (15.29)		1.75 (1.16)	
Education level	Primary education	55.89 (11.46)	0.35 **	1.71 (1.50)	0.44 ****
	Secondary education	50.50 (22.28)		2.63 (1.18)	
	University degree	60.44 (16.77)		2.75 (1.21)	
Previous breast surgery	No	56.50 (17.79)	0.66 *	2.40 (1.15)	0.63 ***
	Yes	54.08 (14.35)		2.63 (1.40)	
Localization of primary tumor	Upper-outer	56.61 (15.89)	0.79 **	2.58 (1.21)	0.35 ****
	Upper-inner	58.33 (11.69)		2.80 (1.30)	
	Lower-outer	53.00 (5.56)		3.00 (0.00)	
	Lower-inner	51.80 (12.29)		1.00 (0.00)	
	Central	47.40 (32.62)		1.50 (0.57)	
Stage	0	43.50 (7.78)	0.65 **	1.00 (0.00)	0.80 ****
	I	58.67 (25.66)		2.00 (1.15)	
	IIA-B	53.96 (15.92)		2.19 (1.17)	
	IIIA-B	57.56 (8.08)		2.00 (1.41)	
Type of mastectomy	Skin-sparing type I–II	50.71 (15.50)	0.35 **	1.91 (1.13)	0.07 ****
	Skin-sparing type IV	62.00 (14.85)		2.80 (0.83)	
	Nipple skin-sparing	61.00 (20.03)		3.22 (0.97)	
	Radical mastectomy	56.44 (16.85)		2.13 (1.35)	
Breast reconstruction timing	Immediate	56.60 (15.28)	0.60 *	2.55 (1.17)	0.11 ***
	Delayed	47.50 (31.77)		1.00 (0.00)	
Type of reconstruction	Expander-prosthesis	53.33 (15.25)	0.82 **	2.44 (0.88)	0.92 ****
	Becker expander	58.67 (17.91)		2.25 (1.16)	
	Latissimus dorsi muscle flap	58.30 (8.73)		2.40 (1.51)	
	Matrix acellular	58.60 (13.66)		2.80 (1.64)	
	Direct prosthesis	51.00 (28.67)		2.50 (1.37)	
Lymph node management	None	52.92 (17.20)	0.20 **	2.45 (1.26)	0.44 ****
	Sentinel lymph node biopsy	57.59 (16.26)		2.44 (1.26)	
	Axillary lymph node dissection	73.50 (6.36)		2.33 (1.17)	
Adjuvant Radiotherapy	No	55.32 (20.71)	0.78 *	2.47 (1.26)	0.92 ***
	Yes	56.57 (9.99)		2.43 (1.15)	
Chemotherapy	No	56.89 (19.73)	0.94 **	2.18 (1.16)	0.99 ****
	Neo-adjuvant	55.08 (21.01)		2.20 (1.30)	
	Adjuvant	55.37 (10.79)		2.22 (1.20)	
Breast symmetrisation	No	57.82 (22.55)	0.55 *	2.17 (1.19)	
	Yes	54.81 (13.15)		2.62 (1.20)	
Evolution	Successful	55.29 (17.44)	0.83 *	2.69 (1.18)	0.39 ***
	Reoperation	56.29 (16.67)		2.30 (1.21)	

* *p*-value obtained by t-Student; ** *p*-value obtained by ANOVA; *** *p*-value obtained by Mann Whitney U; **** *p*- value obtained by Kruskal-Wallis.

**Table 8:** Correlation between the score on the satisfaction with results scales and patient’s characteristics.

		Correlation	*p*
Satisfaction with the breasts	Age	0.007	0.96 *
	BMI	−0.12	0.40 *
	Tumor size	−0.09	0.51 **
	Implant size	−0.16	0.24 **
	Reoperations	0.07	0.61 **
	Years between surgery and the questionnaire	−0.07	0.59 **
Satisfaction with the reconstruction of the NAC	Age	−0.22	0.20 *
	BMI	−0.16	0.35 *
	Tumor size	−0.20	0.27 *
	Implant size	−0.07	0.66 *
	Reoperations	−0.22	0.20 *
	Years between surgery and the questionnaire	−0.40	0.02 *

* *p*-value obtained by Pearson’s correlation coefficient; ** *p*-value obtained by Spearman’s correlation coefficient. NAC = Nipple Areola Complex.

**Table 9:** Differences between satisfaction with care scales mean scores and most important qualitative variables studied.

Variables		Satisfaction with Information	*p*	Satisfaction with the Surgeon	*p*	Satisfaction with Surgical Team	*p*	Satisfaction with Hospital	*p*
Education level	Primary education	63.37 (14.93)	0.34 **	81.33 (35.31)	0.38 **	72.33 (32.91)	0.02 **	60.89 (27.32)	0.003 **
	Secondary education	62.08 (14.91)		92.25 (11.34)		88.00 (17.90)		87.58 (19.32)	
	University degree	72.50 (22.30)		96.44 (10.34)		96.44 (9.93)		91.75 (15.16)	
Previous breast surgery	No	68.75 (18.76)	0.75 *	90.38 (20.37)	0.40 *	87.41 (22.12)	0.96 *	80.17 (25.84)	0.52 *
	Yes	63.92 (18.22)		96.08 (8.23)		91.92 (12.57)		86.31 (15.96)	
Stage	0	69.00 (0.00)	0.84 **	43.00 (60.81)	0.000 **	35.00 (49.50)	0.001 **	36.50 (51.62)	0.03 **
	I	72.00 (19.74)		95.44 (13.67)		94.67 (12.97)		89.78 (16.28)	
	IIA-B	65.64 (18.98)		95.07 (9.98)		90.11 (15.88)		80.59 (22.84)	
	IIIA-B	65.78 (16.81)		87.33 (18.36)		87.11 (18.32)		84.89 (20.26)	
Type of mastectomy	Skin-sparing type I–II	66.06 (18.09)	0.14 **	89.32 (24.24)	0.26 **	88.37 (23.78)	0.78 **	77.58 (30.01)	0.77 **
	Skin-sparing type IV	67.33 (18.00)		98.67 (3.22)		92.00 (15.60)		88.67 (14.77)	
	Nipple skin-sparing	80.11 (20.78)		95.44 (13.66)		93.67 (12.85)		88.89 (14.89)	
	Radical mastectomy	62.00 (16.20)		90.31 (15.93)		84.69 (20.73)		80.13 (21.72)	
Breast reconstruction timing	Immediate	68.80 (18.69)	0.04 *	91.87 (18.65)	0.59 *	88.24 (20.53)	0.64 *	82.00 (24.05)	0.65 *
	Delayed	52.50 (8.02)		91.75 (11.78)		92.50 (15.00)		78.67 (19.14)	
Type of reconstruction	Expander-prosthesis	70.00 (14.45)	0.48 **	91.33 (25.94)	0.04 **	85.53 (28.86)	0.70 **	74.27 (28.46)	0.54 **
	Becker expander	66.00 (17.40)		90.92 (15.13)		87.69 (16.56)		80.92 (26.49)	
	Latissimus dorsi muscle flap	62.30 (18.80)		85.90 (17.34)		89.30 (16.37)		86.00 (19.15)	
	Matrix acellular	78.20 (22.16)		96.40 (8.05)		96.40 (4.93)		84.60 (16.05)	
	Direct prosthesis	64.86 (25.91)		100.00 (0.00)		94.43 (14.74)		92.14 (14.49)	
Breast symmetrisation	No	64.94 (21.48)	0.38 *	92.61 (16.41)	0.40 *	92.89 (13.90)	0.35 *	86.00 (25.38)	0.13 *
	Yes	68.94 (16.84)		91.44 (19.24)		88.16 (22.65)		79.35 (22.61)	
Evolution	Successful	73.38 (21.53)	0.11 *	90.95 (22.70)	0.65 *	87.91 (24.09)	0.49 *	80.77 (28.78)	0.72 *
	Reoperation	63.04 (14.88)		92.57 (13.90)		89.11 (16.67)		82.63 (18.97)	

* *p*-value obtained by Mann Whitney U; ** *p*-value obtained by Kruskal-Wallis.

## Data Availability

Database is available on “Castro de Luna, Gracia; Garcia, Silvia (2021)”, “BREAST QUESTIONNAIRE SILVIA GARCIA”, Mendeley Data, V1, doi:10.17632/c4vnc6tcx3.1.
